# Microvesicles carrying LRP5 induce macrophage polarization to an anti‐inflammatory phenotype

**DOI:** 10.1111/jcmm.16723

**Published:** 2021-07-19

**Authors:** Aureli Luquero, Gemma Vilahur, Javier Crespo, Lina Badimon, Maria Borrell‐Pages

**Affiliations:** ^1^ Cardiovascular Program ICCC IR‐Hospital de la Santa Creu i Sant Pau IIB‐Sant Pau Barcelona Spain; ^2^ CIBER‐CV Instituto de Salud Carlos III Madrid Spain; ^3^ Cardiovascular Research Chair UAB Barcelona Spain

**Keywords:** inflammation, lipids, LRP5, macrophages, MV

## Abstract

Microvesicles (MV) contribute to cell‐to‐cell communication through their transported proteins and nucleic acids. MV, released into the extracellular space, exert paracrine regulation by modulating cellular responses after interaction with near and far target cells. MV are released at high concentrations by activated inflammatory cells. Different subtypes of human macrophages have been characterized based on surface epitopes being CD16^+^ macrophages associated with anti‐inflammatory phenotypes. We have previously shown that low‐density lipoprotein receptor‐related protein 5 (LRP5), a member of the LDLR family that participates in lipid homeostasis, is expressed in macrophage CD16^+^ with repair and survival functions. The goal of our study was to characterize the cargo and tentative function of macrophage‐derived MV, whether LRP5 is delivered into MV and whether these MV are able to induce inflammatory cell differentiation to a specific CD16^−^ or CD16^+^ phenotype. We show, for the first time, that lipid‐loaded macrophages release MV containing LRP5. LDL loading induces increased expression of macrophage pro‐inflammatory markers and increased release of MV containing pro‐inflammatory markers. Conditioning of fresh macrophages with MV released by *Lrp5*‐silenced macrophages induced the transcription of inflammatory genes and reduced the transcription of anti‐inflammatory genes. Thus, MV containing LRP5 induce anti‐inflammatory phenotypes in macrophages.

## 1 INTRODUCTION

Extracellular microvesicles (MV) are cell shed particles (diameter ranging from 0.1 to 1 μm) released when cells are stimulated, damaged or undergoing apoptosis.^1^, ^2^, ^3^, ^4^, ^5^, ^6^, ^7^, ^8^, ^9^ They can also be released by healthy cells.^10^ MV are formed by direct budding of small cytoplasmatic protrusions that are detached from the cell surface into the extracellular space. They are characterized by the externalization of the procoagulant anionic phosphatidylserine making MV Annexin V positive.^11^, ^12^ Their cargo defines their shape, size and function. Because MV reflect the condition of their parental cells, they represent a potential diagnostic tool to identify diverse diseases, including cancer, metabolic and cardiovascular diseases. When MV are shed from their cells of origin, they circulate in blood carrying messengers for recipient cells that will receive the signal and regulate their cellular growth, differentiation and transformation.^4^, ^5^, ^6^, ^7^, ^8^, ^9^, ^10^, ^13^, ^14^, ^15^, ^16^ Indeed, the interaction of MV with target cells and the release of their content modulate cell responses.^17^ MV have been described in inflammatory processes and associated with several cardiovascular risk factors^18^ contributing to the initiation and progression of cardiovascular diseases, including atherosclerosis.

Atherosclerosis is characterized by chronic inflammation induced by increasing accumulation of low‐density lipoproteins (LDL) and apoptotic cells in the intima layer of the arteries.^19^ The low‐density lipoprotein receptor‐related protein 5 (LRP5) is a multifunctional receptor involved in both endocytosis of lipids and the canonical Wnt signalling pathway.^20^ LRP5 is a single‐pass transmembrane receptor that participates in the Wnt/β‐catenin signalling pathway. LRP5 activation causes the stabilization of β‐catenin that translocates into the nucleus, binds to the transcription factor TCF/LEF1 and starts the transcription of Wnt target genes that regulate fundamental aspects of embryonic cell development^21^ and adult cell function.^20^, ^22^, ^23^

LDL loading induces high LRP5 expression in human macrophages.^20^ Macrophages can be classified into classical activated CD14^+^CD16^−^, pro‐inflammatory macrophages and alternatively activated CD14^−^CD16^+^, anti‐inflammatory macrophages.^24^, ^25^ LRP5 participates in inflammation and macrophage polarization by association with the anti‐inflammatory macrophage subtype CD16^+^ derived from CD14^+^CD16^+^ patrolling circulating monocytes.^26^ LRP5 confers the motile function to CD16^+^ macrophages by triggering the canonical Wnt signalling. Furthermore, CD16^+^LRP5^+^ macrophages, found in advanced atherosclerotic human plaques, trigger an anti‐inflammatory, defensive and repair response.^26^

The in‐depth understanding of the formation, cargo and function of MV is an ongoing task in the field. The objectives of this study were (a) to characterize the cargo and function of macrophage‐derived MV and their ability to induce inflammatory cell differentiation to a CD16^−^ or a CD16^+^ phenotype, and (b) to investigate whether LRP5 is delivered into MV and whether it can exert paracrine functions.

We show that LDL‐loaded macrophages release MV carrying LRP5 and exert paracrine and/or autocrine regulation. LDL loading induces increased expression of macrophage cellular pro‐inflammatory markers and increased release of MV. Interestingly, LRP5 is released in MV that contain both pro‐ and anti‐inflammatory markers. Conditioning of recipient macrophages with MV released by *Lrp5*‐silenced macrophages induced pro‐inflammatory gene transcription and a reduced expression of anti‐inflammatory genes indicating that LRP5 induces macrophage differentiation into the anti‐inflammatory phenotype.

## 2 METHODS

### 2.1 Isolation of human monocytes and human macrophages primary cultures and LDL loading

Human monocytes were obtained by standard protocols from buffy coats of healthy blood donors.^20^, ^26^, ^27^, ^28^ All procedures were approved by the Institutional Review and Ethics Committee, and the investigation conformed to the principles outlined in the Declaration of Helsinki with informed consent given by donors. Briefly, blood was applied on 15 mL of Ficoll‐Hypaque and centrifuged at 300 g for 1 hour at 22°C, with no brake. Mononuclear cells were obtained from the central white band of the gradient, exhaustively washed in Dulbecco's phosphate buffer saline, and suspended in RPMI medium (Gibco) supplemented with 10% human serum AB (Sigma). Isolated monocytes (Mo) were left overnight in culture, washed and treated with 100 μg/mL nLDL (native LDL) or agLDL (aggregated LDL) for the described times. A second set of isolated Mo were left 7 days in culture and allowed to differentiate into macrophages (Mac) by changing the cell culture media (RPMI supplemented with 10% human serum AB, 100 units/mL penicillin and 100 µg/mL streptomycin) every 3 days. After several washings with PBS to completely remove serum, human macrophages were incubated with 100 μg/mL nLDL or 100 µg/mL agLDL in serum‐free medium.^20^, ^26^, ^27^, ^28^ At the end of the experiments, human Mo and Mac were exhaustively washed (twice with PBS, twice with PBS/1% BSA, once with PBS/1%BSA/heparin 100 U/mL, twice with PBS/1% BSA and twice with PBS) and prepared for the collection of mRNA and protein detection as described below.

### 2.2 LDL isolation and modification

Human LDL (d1.019‐d1.063 g/mL) were obtained as previously described.^28^ Briefly, human LDLs were obtained from pooled sera of normocholesterolemic volunteers and isolated by sequential ultracentrifugation. LDLs were dialyzed three times against 200 volumes of 150 mmol/L NaCl, 1 mmol/L EDTA, and 20 mmol/L Tris‐HCl, pH 7.4, overnight and once against 150 mmol/L NaCl. LDL protein concentration was determined by the bicinchoninic acid, and vortexing was monitored by measuring the turbidity (absorbance at 680 nm). The model system of agLDL was generated by vortexing LDL (1 mg/mL) for 4 minutes at room temperature at maximal speed. The percentage of LDL in aggregated form was calculated by measuring the fraction of protein recovered in the pellet obtained after centrifugation at 10 000 *g* for 10 minutes. The different fractions were analysed by agarose electrophoresis, and the precipitated fraction composed of 100% agLDL was added to cell cultures.

### 2.3 MV isolation and quantification

LDL‐loaded or non‐loaded human Mo and Mac were cultured for 24 or 48 hours and the MV released into the supernatants collected. MV were isolated by five‐step high‐speed centrifugations. Briefly, 2 mL of fresh supernatant aliquots were centrifuged at 3200 *g* for 20 minutes to guarantee complete cell and debris removal. The recovered supernatants were centrifuged at room temperature at 300 *g*, (10 minutes); at 1200 *g*, (20 minutes); and at 12 500 *g* (5 minutes) in two repeated processes to ensure the elimination of nLDL or agLDL. The cleared supernatants were transferred to another vial and centrifuged at 20 500 *g* for 150 minutes at RT to pellet the MV. Supernatants were removed and the MV‐enriched pellets (MVp) were suspended in 100 μL citrate‐PBS.

MVp (5 μL) in combination with 2‐3 specific monoclonal antibodies (1‐5 μL each) labelled with phycoerythrin, 488 or the isotype‐matched control antibodies were added in a final volume of 50 μL annexin binding buffer with 5 μL of Annexin V (AV) to label and characterize AV^+^MV with bioactive and biomarker molecules from their parental cells. Table S1 shows the different antibodies and the concentrations used for microvesicle identification and characterization. Samples were incubated 20 minutes at room temperature in the dark and diluted with annexin binding buffer before being immediately analysed and counted on a FACSCanto II flow cytometer. The number of monocytes or macrophage per well were counted using Neubauer chambers, and the number of MV/cell type was obtained.

AV binding level was corrected for autofluorescence using fluorescence signals obtained with MV in a calcium‐free buffer PBS. MV were identified and quantified based on their forward scatter/side scatter characteristics according to their size, binding or not to AV and reactivity to specific monoclonal antibodies. Figure S1 shows representative plots for MV identification and characterization by flow cytometry analysis.

Acquisition was performed at 1 minute per sample and flow rate was measured before each experiment. Forward scatter, side scatter and fluorescence data were obtained with the settings in the logarithmic scale. The lower detection limit was placed as a threshold above the electronic noise of the flow cytometer. To identify positive marked events, thresholds were also set based on samples incubated with the same final concentration of isotype‐matched control antibodies after titration experiments. Data were analysed with the FACSDivaTM software (version 6.1.3; Becton Dickinson). To reduce background noise, buffers were prepared on the same day and filtered through 0.2 μm pore size filters under vacuum.

### 2.4 Macrophages isolation by flow cytometry

Cellular protein expression was assessed in primary cultures of human macrophages by flow cytometry. Cell suspensions in flow cytometry buffer (0.1% sodium azide/1%BSA/PBS) were gently centrifuged at 200 *g*, 10 minutes, RT. Pellet samples were then suspended in flow cytometry buffer and stained for 20 minutes with specific antibodies as described in Table S2. Figure S2 shows the gating strategy for live macrophages by flow cytometry analysis. Samples were diluted with 400 μL flow cytometry buffer prior to being immediately analysed. For each sample, at least 10 000 events were acquired on a FACSCantoII (Beckton Dickinson). Data was analysed with the FACSDiva 6.1.3 software.

### 2.5 Macrophages isolation by cell sorter

Lipid loaded macrophages were gently detached from culture dishes and stained with CD11b, CD14 and CD206 or CD80 antibodies (Table S2) for 30 minutes in 100 µL 0.5%BSA/PBS. The reaction was stopped by adding 4 volumes of 0.5%BSA/PBS to the mix. Cells were sorted using a FACSAria‐I (BD Biosciences) operated using a 100 µm nozzle with the 488 nm and 633 nm laser lines. After positive selection of CD11b^+^CD14^+^ cells, two populations were sorted: CD11b^+^CD14^+^CD206^+^/CD11b^+^CD14^+^CD206^−^ or CD11b^+^CD14^+^CD80^+^/CD11b^+^CD14^+^CD80^−^. After sorting, macrophage populations were centrifuged separately for 10 minutes at 200 *g*. Then, cells were suspended in RPMI GlutaMax medium supplemented with 10% AB human serum with 1% penicillin/streptomycin and seeded into 6‐well plates for 24 hours. Flow cytometry data acquisition, analysis and image preparation were performed using the FACSDiva software (BD Bioscience).

### 2.6 Supernatant collection

Cell sorted macrophages were cultured in serum‐free RPMI GlutaMax medium for 2 days when supernatants were collected and centrifuged at 15 000 *g*, 15 minutes, 4°C. Pellets were discharged and supernatants were precipitated using a methanol/chloroform protocol. Briefly, one volume of supernatant was mixed with three volumes of cold methanol and one volume of chloroform, vortexed vigorously for 30 seconds, and then, three volumes of H_2_O were added to the sample to induce phase separation. The mix was centrifuged at 10 000 *g* for 5 minutes, and the upper phase was eliminated without disturbing the interphase. Three volumes of methanol were added to the mix, and samples were centrifuged at 10 000 *g* for 5 minutes. Supernatants were discharged and the precipitated proteins (pellet) were let to air‐dry. Finally, samples were suspended in 100 µL of lysis protein buffer solution and frozen at −20°C until western blots were performed.

### 2.7 Western blot

Protein extracts (50 µL) were resolved by SDS‐PAGE and transferred to nitrocellulose membranes, blocked with 5% bovine serum albumin and probed for monoclonal primary antibodies against IL‐1β, TNFα and TGF‐β from Cell Signalling. Membranes were then washed and blotted with antimouse secondary antibodies (Dako). Band densities were determined with the ChemiDoc XRS system (Bio‐Rad) in chemiluminescence detection modus and Quantity‐One software (Bio‐Rad).

### 2.8 LRP5 silencing

Human macrophages were transfected with 100 nmol/L of siRNA‐Random (siR) or siRNA‐LRP5 (si5) using HiPerfect^®^ as recommended by the manufacturer. Small anti‐LRP5 interfering RNAs (si5, s8293) were synthesized by Applied Biotechnologies and Silencer Selective Negative Control #1 (siR, 4390843) by Ambion.

### 2.9 RNA isolation and Real time PCR

Total RNA was isolated from cultured human monocytes and macrophages using the total RNA extraction kit (Qiagen). Total RNA concentration was determined by NanoDrop ND‐1000 spectrophotometer (NanoDrop Technologies, Inc), and purity was checked by the A260/A280 ratio (ratios between 1.8 and 2.1 were considered acceptable), in addition, an agarose gel was run to assess quality. cDNA was synthesized from 1 μg RNA with cDNA reverse transcription kit (Qiagen) The resulting cDNA samples were amplified by polymerase chain reaction (PCR) using a DNA thermal cycler (MJ Research) and the following specific human probes from Applied Biotechnologies: LRP5, iNOS, CD80, CD163 and IL1Ra. Normalization was performed against r18S.

### 2.10 Statistical analysis

A StatView statistical package was used for all the analysis. Results are expressed as mean ± SD or n (%) when indicated. When possible, comparisons among groups were performed by parametric (one factor ANOVA) analysis. Statistical significance was considered when *P* < .05. All the experiments were performed at least three times.

## 3 RESULTS

### 3.1 Monocytes and macrophages induce LRP5+MV secretion

**FIGURE 1:**
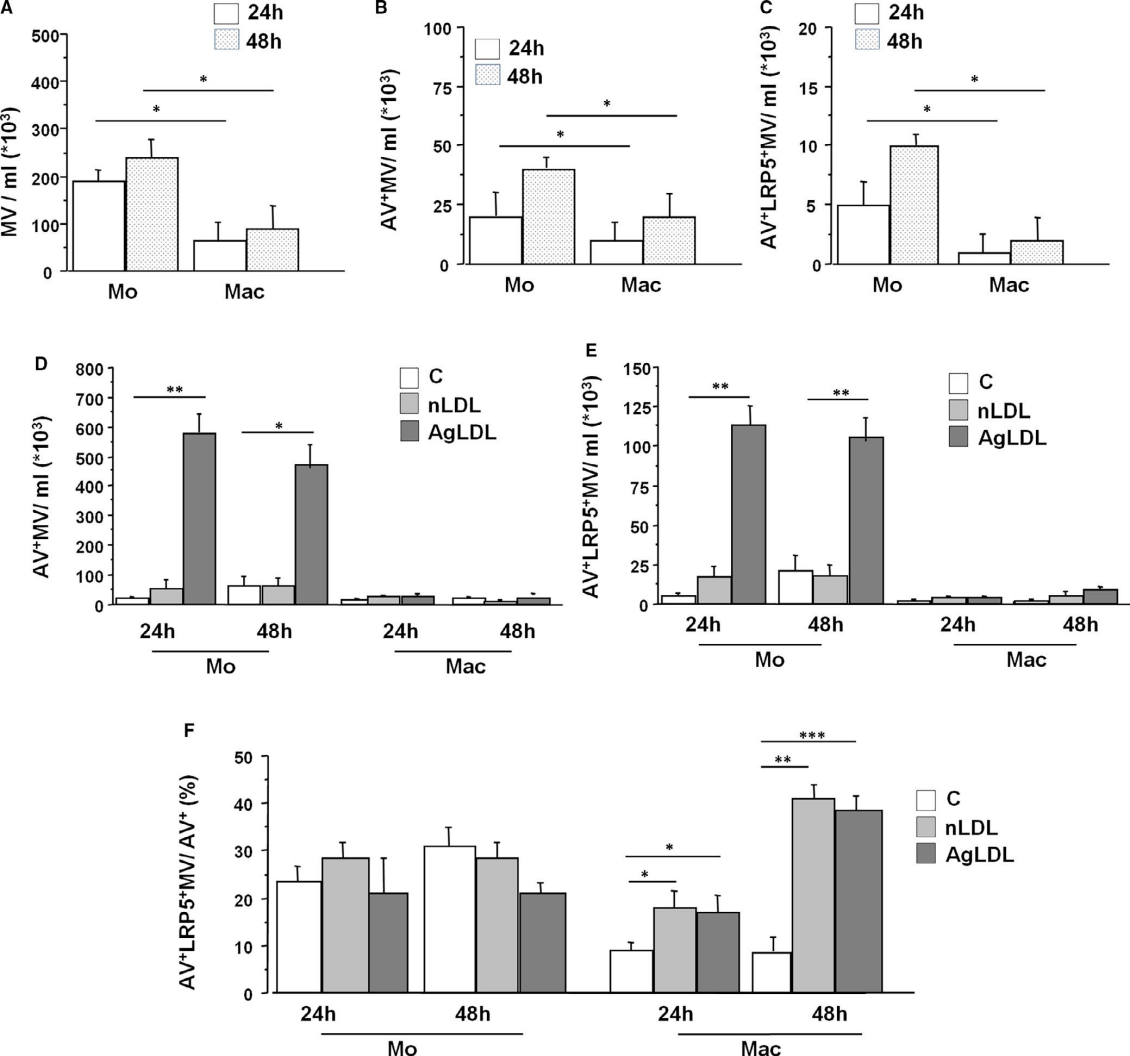
AgLDL treatments in macrophages induce LRP5^+^MV secretion. 24 hours or 48 hours supernatants from undifferentiated monocytes (Mo) or from 7 to 10 days fully differentiated macrophages (Mac) were collected and the amount of (A) microvesicles/mL; (B) Annexin V^+^ microvesicles/mL and (C) Annexin V^+^ LRP5^+^ microvesicles/mL were analysed. (D) Monocytes (Mo) or macrophages (Mac) were treated with 100 μg/mL nLDL or 100 μg/mL agLDL for 24 hours or 48 hours and the amount of AV^+^ MV/mL and of (E) AV^+^ LRP5^+^ MV/mL was analysed. (F) The ratio between MV that are AV^+^LRP5^+^/AV^+^ in control and lipid‐loaded monocytes (Mo) and macrophages (Mac). All experiments were performed at least four times in duplicates or triplicates. *P < .05, **P < .01, ***P < .005

To characterize the released MV, supernatants of primary cultures of monocytes and macrophages were collected after 24 hours and 48 hours (Figure 1A). Mo release around 200 000 MV/mL after 24 hours and around 250 000 MV/mL after 48 hours in culture (Figure 1A). Mac release around 52 000 MV/mL after 24 hours and almost 100 000 MV/mL after 48 hours in culture (Figure 1A). These time differences in MV release did not reach statistical significance. However, there was a statistically significant difference in MV release between Mo and Mac both at 24 hours and at 48 hours (Figure 1A).

We then analysed released Annexin V‐positive MV (AV^+^MV) and no significant differences were found between MV release at 24 hours and 48 hours neither in Mo nor in Mac. Mo released higher number of AV^+^MV than Mac both at 24 hours and at 48 hours (Figure 1B). AV^+^MV released by monocytes and by macrophages at 24 hours and 48 hours contained LRP5 (Figure 1C).

### 3.2 Lipid loading increases LRP5^+^MV secretion

We have previously shown that lipid loading with modified lipoproteins (agLDL) increases LRP5 expression in macrophages.^20^, ^26^ We hypothesized that the LDL loading would increase the generation of MV carrying LRP5. Primary cultures of human monocytes and macrophages were treated with 100 μg/mL nLDL or agLDL for 24 hours or 48 hours and, indeed, agLDL loading induced a massive generation of AV^+^MV from monocytes while a modest amount of AV^+^MV were released by macrophages (Figure 1D). LDL loading induced the release of AV^+^LRP5^+^MV in larger quantities in monocytes than in macrophages (Figure 1E). However, the relative release of AV^+^LRP5^+^MV (normalized by total AV^+^MV) was significantly induced by LDL loading in macrophages both after 24 hours and 48 hours incubation (Figure 1F).

We then estimated the amount of MV produced by each monocyte or macrophage (MV/Mo and MV/Mac). Lipid‐loaded Mo release more AV^+^ MV than Mac after 24 hours (175 ± 21 AV^+^MV/Mo vs 6 ± 0.8 AV^+^MV/Mac, Figure S3A) and 48 hours agLDL incubation (108 ± 15 AV^+^MV/Mo vs 6 ± 0.4 AV^+^MV/Mac, Figure S3A). AgLDL treatments induced more LRP5^+^MV release in individual monocytes than in macrophages both after 24 hours and 48 hours incubation (Figure S3B). Finally, the relative amount of AV^+^LRP5^+^MV/cell type (normalized by AV^+^MV/cell type) released by macrophages was higher than that released by monocytes after 24 hours and 48 hours agLDL incubation (Figure S3C).

### 3.3 agLDL loading induces macrophage polarization

**FIGURE 2:**
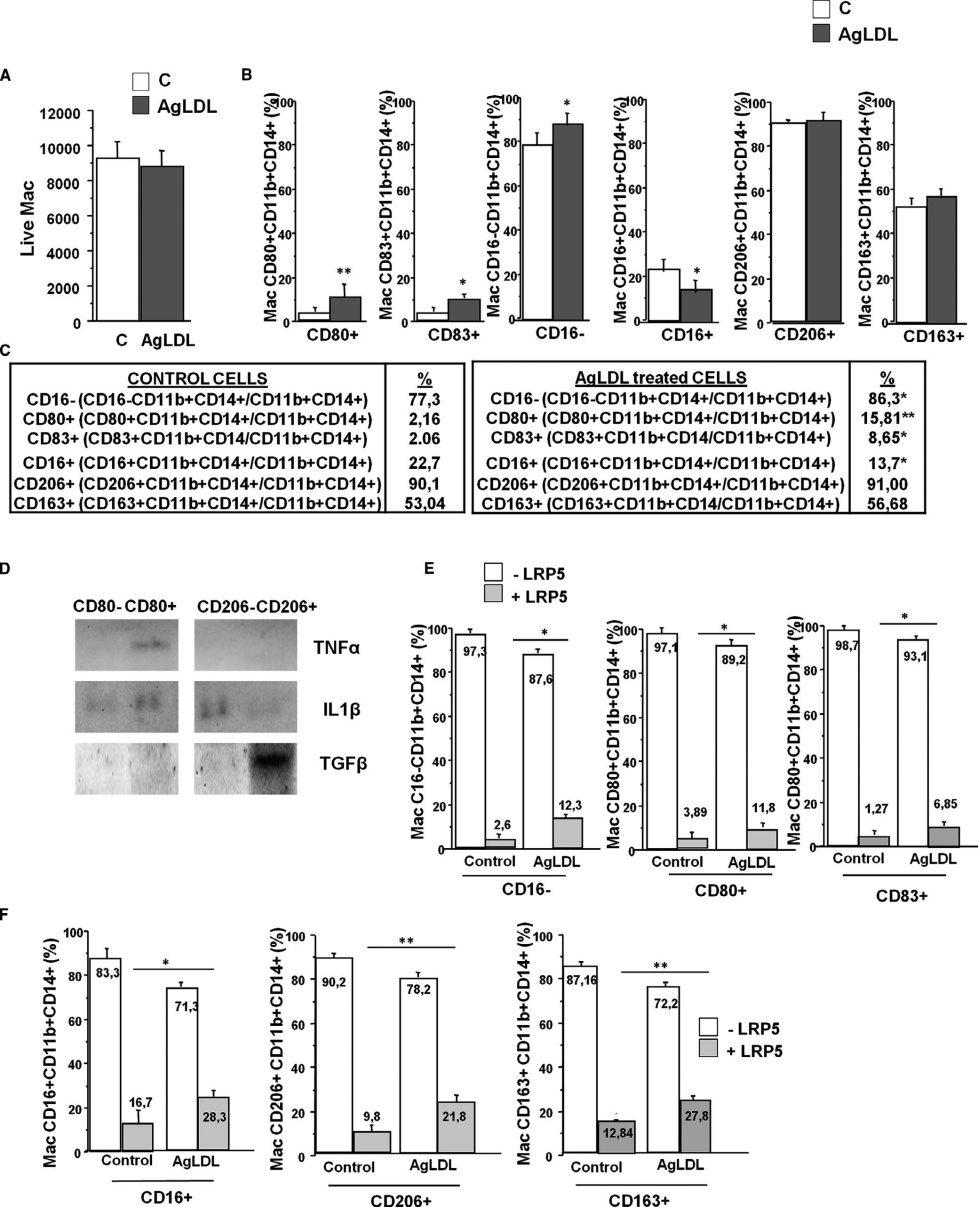
AgLDL treatments induce LRP5 expression in pro‐ and anti‐inflammatory macrophages. (A) Flow cytometry was used to quantify live macrophages after treatment or not with 100 μg/mL agLDL. (B) Cellular expression of CD80, CD83, CD16, CD206 and CD163 in control and agLDL‐treated macrophages. (C) Quantification of the graphs depicted in (B). (D) TNFα, IL1β and TGFβ expression in supernatants of agLDL‐treated and cell sorted macrophage subpopulations. (E) LRP5 expression levels by flow cytometry in CD16^−^, CD80^+^ and CD83^+^ pro‐inflammatory macrophages in control and after agLDL treatment. (F) Same in anti‐inflammatory CD16^+^, CD2016^+^ and CD163^+^ expressing macrophages. All experiments were performed at least four times in duplicates or triplicates. *P < .05, **P < .01

We previously observed that LRP5 is mainly expressed in CD16^+^ macrophages and lipid loading induces LRP5 expression in these cells^20^, ^26^; therefore we investigated whether macrophage polarization could be induced by agLDL. Lipid‐loaded macrophages were gently detached from the culture dish and these live macrophages were counted by flow cytometry showing that the agLDL loading did not affect cell survival (Figure 2A). Macrophage population was defined by size, with specific pro‐ and anti‐inflammatory antibodies and with the well‐known macrophage markers CD11b and CD14. Figure S2 shows the gating strategy for live macrophages by flow cytometry analysis. Results show that lipid loading induces the expression of CD80^+^ and CD83^+^ and reduces the expression of cell surface CD16 (CD80^+^: 2.16% expression in control conditions to 15.81% expression after agLDL loading; CD83^+^: 2.06% expression in control conditions to 8.65% expression after lipid loading and CD16^−^: 77.3% expression in control conditions and 86.3% expression in lipid loaded cells). The expression levels of the anti‐inflammatory marker CD16^+^ was reduced in lipid loaded macrophages while the expression levels of the anti‐inflammatory proteins, CD206^+^ and CD163^+^ did not vary with respect to control conditions. Therefore, lipid loading induces a pro‐inflammatory polarization in macrophages with increased CD80^+^, CD83^+^ and CD16^−^ expression in cells (Figure 2B,C).

Macrophage pro‐inflammatory phenotype after lipid loading was confirmed by pro and anti‐inflammatory protein secretion analyses. Cell sorting was performed on lipid loaded macrophages to obtain CD11b^+^CD14^+^CD206^−^ and CD11b^+^CD14^+^CD206^+^ or CD11b^+^CD14^+^CD80^−^ and CD11b^+^CD14^+^CD80^+^ macrophage subpopulations. The different macrophage subpopulations were seeded in culture dishes and supernatants were collected after 48 hours. Increased release of the pro‐inflammatory proteins TNFα and IL1β was observed in the pro‐inflammatory CD80^+^ subpopulation while the levels remained low in the anti‐inflammatory CD206^+^ subpopulation (Figure 2D). Conversely, the release of the anti‐inflammatory protein TGFβ was higher in the CD206^+^ macrophage subpopulation than in the CD80^+^ macrophage subpopulation (Figure 2D). Therefore, inflammatory protein release confirms the pro‐inflammatory polarized phenotype in macrophages observed by cell surface markers expression after lipid loading.

### 3.4 Lipid loading induces LRP5 expression in macrophages

We next examined the expression levels of LRP5 in the different macrophage subpopulations by staining macrophages with a specific antibody for LRP5. Pro‐inflammatory CD16^−^ macrophages show increased LRP5 cellular expression after agLDL loading compared to controls (12.3% and 2.6% respectively; Figure 2E). Similarly, CD80^+^ and CD83^+^ macrophages showed increased levels of cellular LRP5 after lipid loading compared to controls (CD80^+^: 3.89% expression in control conditions to 11.8% expression after lipid loading; CD83^+^: 1.27% expression in control conditions to 6.85% expression after agLDL loading; Figure 2E). Interestingly, macrophages that express the anti‐inflammatory markers CD16^+^, CD206^+^ or CD163^+^ also showed significantly increased LRP5 expression after agLDL loading (CD16^+^: 16.7% in control conditions to 28.3% in lipid‐loaded macrophages, CD206^+^: 9.8% in controls to 21.8% in lipid‐loaded macrophages and CD163^+^: 12.84% in controls to 27.8% in agLDL‐treated macrophages) indicating that lipid loading induce LRP5 expression in both pro‐ and anti‐inflammatory macrophages but with a higher expression in anti‐inflammatory macrophages (Figure 2F).

### 3.5 Inflammatory profile of MV after lipid loading

**FIGURE 3:**
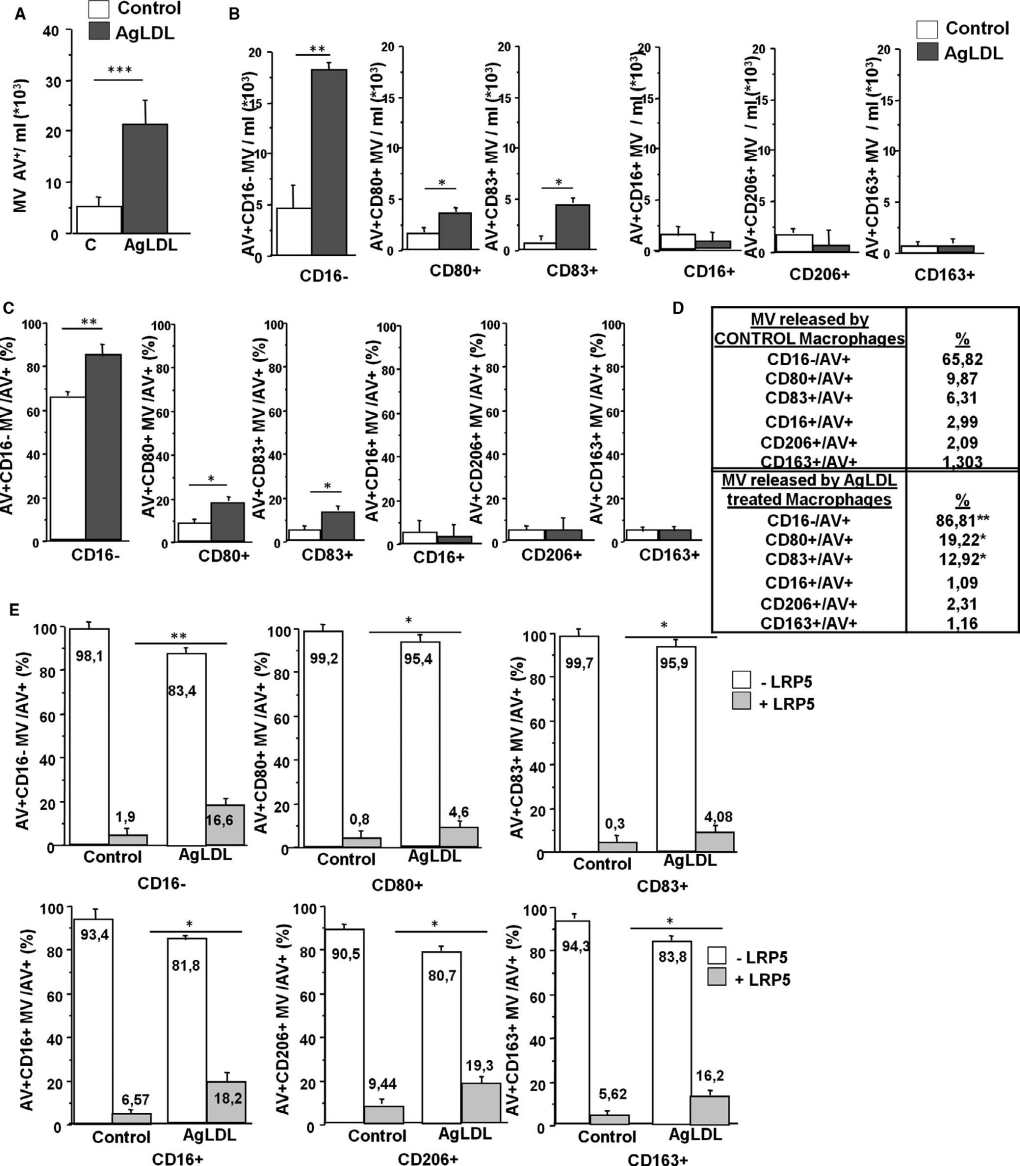
Macrophages treated with agLDL release LRP5^+^MV. (A) Flow cytometry was used to quantify AV^+^ MV secreted by control and 100 μg/mL AgLDL‐treated macrophages. (B) CD16^−^ MV/mL, CD80^+^ MV/mL, CD16^+^ MV/mL and CD206^+^ MV/mL released by control and agLDL‐treated macrophages (C) Same as in (B) but performing the ratio against AV^+^MV. (D) Quantification of the graphs shown in (C). (E) Flow cytometry detection of LRP5 expression in CD16^−^MV, CD80^+^MV, CD16^+^MV and CD206^+^MV released by control and agLDL‐treated macrophages. All experiments were performed at least four times in duplicates or triplicates. *P < .05, **P < .01, ***P < .005

MV release was investigated in supernatants from LDL‐loaded macrophage (100 μg/mL agLDL). Lipid loading induced significantly higher release of AV^+^MV (Figure 3A). LDL loading induced a significant increase in the release of CD16^−^, CD80^+^ and CD83^+^ MV but not CD16^+^, CD206^+^ and CD163^+^ MV (Figure 3B). Normalization by total AV^+^ MV showed that only CD16^−^, CD80^+^ and CD83^+^ MV levels were increased after agLDL loading (CD16^−^: 65.82% in control conditions to 86.81% after lipid loading, CD80^+^: 9.87% in control conditions to 19.22% after lipid loading and CD83^+^: 6.31% in control conditions to 12.92% after lipid loading, Figure 3C,D). Macrophage‐derived MV containing CD16^+^, CD206^+^ and CD163^+^ anti‐inflammatory markers remained constant before and after macrophage lipid loading (CD16^+^: 2.99% in control conditions vs 1.09% in lipid‐loaded macrophages, CD206^+^: 2.09% in control conditions vs 2.31% after agLDL loading and CD163^+^: 1.30% in control conditions to 1.16% after lipid loading Figure 3C,D) indicating that lipid loading induces the release of MV containing pro‐inflammatory markers.

### 3.6 LRP5^+^MV contain pro‐inflammatory and anti‐inflammatory proteins

MV released from lipid‐loaded and non‐loaded macrophages were isolated and stained for pro‐inflammatory and anti‐inflammatory markers and for LRP5. Interestingly, LRP5 was delivered into MV containing both pro‐inflammatory and anti‐inflammatory proteins, indicating that the delivery of LRP5 into MV is independent of the inflammatory proteins delivered into the MV (Figure 3E).

### 3.7 Characterization of donor macrophages and their released MV

**FIGURE 4:**
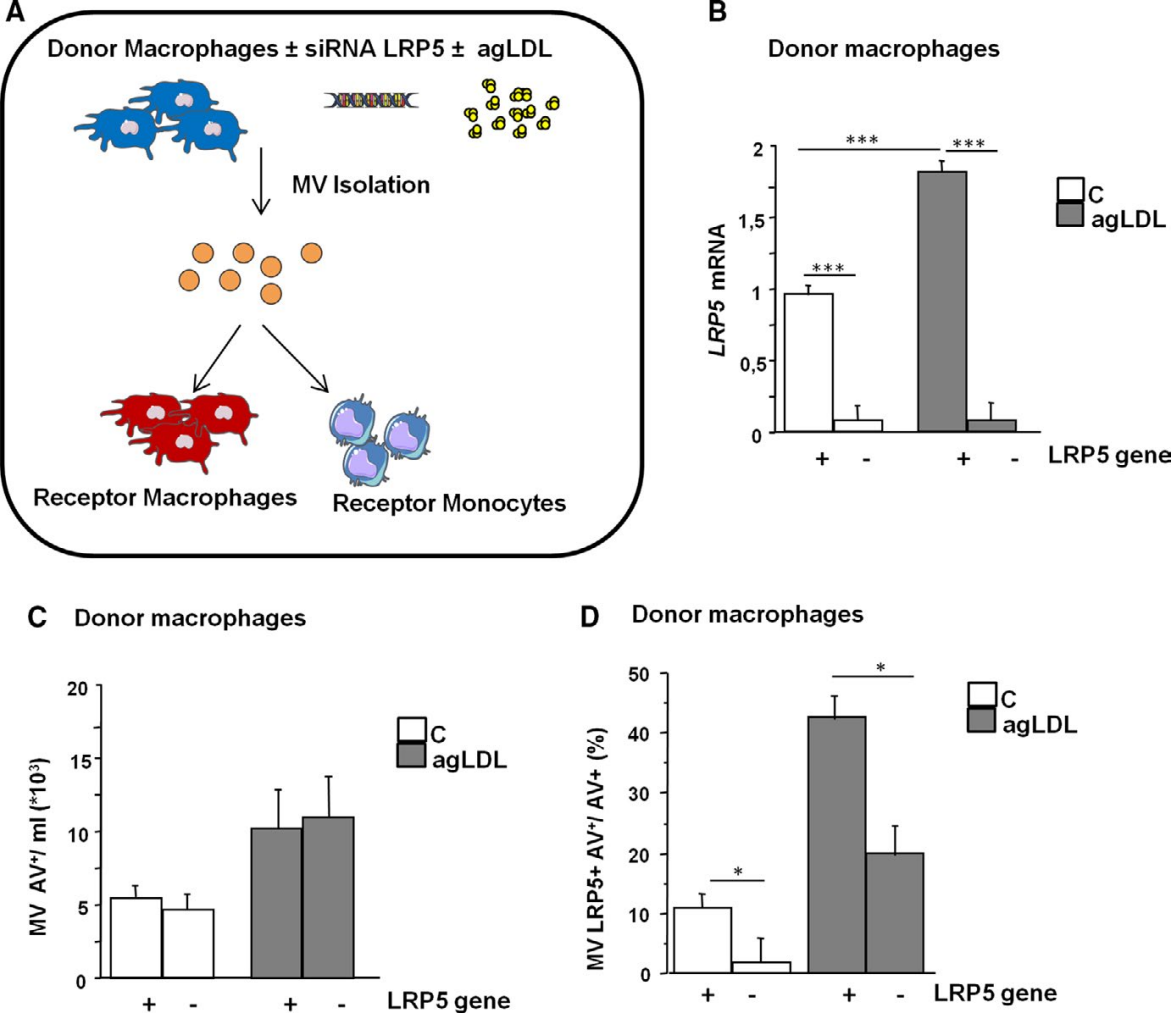
Characterization of donor macrophages and their secreted MV. (A) Schematic of experiment. Donor macrophages were silenced or not for LRP5 (siLRP5) and treated or not with agLDL. After 48 hours, macrophage secreted MV were isolated and suspended to treat receptor macrophages or receptor monocytes. (B) LRP5 gene expression in donor macrophages after LRP5 silencing and agLDL treatments. (C) Macrophage‐derived AV^+^MV/mL secreted by donor macrophages. (D) Macrophage‐derived LRP5^+^MV released by donor macrophages. Experiments were performed four times in triplicates. *P < .05, **P < .01, ***P < .005

Macrophage specific inhibition of LRP5 expression (with siRNA) was used to identify whether LRP5 was playing a role in macrophage differentiation towards a CD16^−^ or a CD16^+^ phenotype. Macrophages were silenced or not for LRP5 and agLDL‐loaded or not (Figure 4A). Analysis of donor macrophages mRNA expression by RT‐PCR showed a 92 ± 3% LRP5 reduction in siRNA‐LRP5 control cells and a 90 ± 2% LRP5 reduction in siRNA‐LRP5 lipid‐loaded macrophages. LRP5 mRNA expression was increased in lipid‐loaded macrophages (Figure 4B). LRP5 silencing did not modify the number of AV^+^MV/mL released by macrophages neither in control nor in lipid‐loaded conditions (Figure 4C). However, a consistent reduction in LRP5^+^AV^+^MV release by siRNA‐LRP5‐treated macrophages was observed both in untreated and agLDL‐loaded macrophages (Figure 4D).

### 3.8 Gene expression levels in conditioned macrophages

**FIGURE 5:**
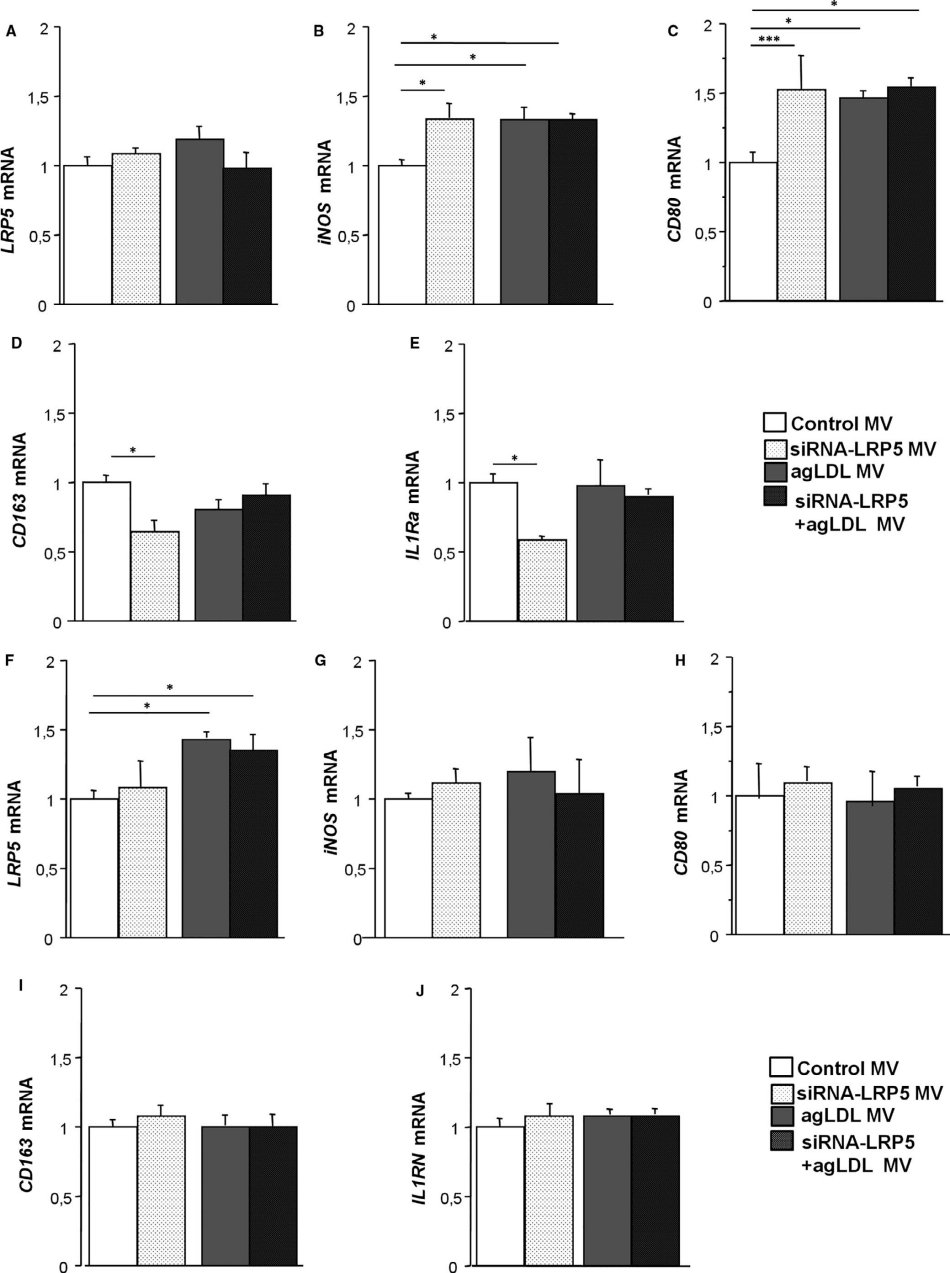
Gene expression levels in receptor macrophages and monocytes. Receptor macrophages were treated with macrophage‐derived MV released by Control macrophages, siRNA‐LRP5‐treated macrophages, 100 μg/mL agLDL‐treated macrophages or siRNA‐LRP5+AgLDL‐treated macrophages and mRNA expression levels of (A) *LRP5*, (B) *iNOS,* (C) *CD80*, (D) *CD163* and (E) *IL1Ra* were analysed. Receptor monocytes were treated with macrophage‐derived MV released by Control macrophages, siRNA‐LRP5‐treated macrophages, 100 μg/mL agLDL‐treated macrophages or siRNA‐LRP5+AgLDL‐treated macrophages and mRNA expression levels of (F) *LRP5*, (G) *iNOS*, (H) *CD80*, (I) *CD163* and (J) *IL1Ra* were analysed. Experiments were performed four times in triplicates. *P < .05, ***P < .005

MV released by the different sets of donor macrophages were isolated and used to condition naive macrophages and monocytes. Treatment of naive macrophages with macrophage‐derived MV released by control macrophages or with MV released by siRNA‐LRP5‐treated macrophages did not modify their LRP5 cellular mRNA expression (Figure 5A). Similarly, treatment with MV released by control or lipid‐loaded macrophages did not modify LRP5 gene expression levels in recipient macrophages indicating that LRP5 contained in MV does not affect LRP5 gene transcription (Figure 5A).

However, macrophage gene transcription of the pro‐inflammatory molecules iNOS and CD80 was increased by MV devoid of LRP5 indicating that LRP5 blocks the expression of pro‐inflammatory genes in recipient macrophages (Figure 5B,C). Treatment with MV released by lipid‐loaded macrophages induced iNOS and CD80 gene transcription in recipient macrophages independent of LRP5 expression (Figure 5B,C). The anti‐inflammatory genes CD163 and IL1Ra showed decreased expression levels in macrophages conditioned with MV released by siRNA‐LRP5 macrophages, indicating that LRP5^+^MV induce higher levels of anti‐inflammatory genes expression in recipient macrophages (Figure 5D,E). Anti‐inflammatory gene transcription in macrophages conditioned with MV released by lipid‐loaded macrophages remained constant independently of LRP5 expression in donor macrophages (Figure 5D,E).

### 3.9 Gene transcription in conditioned monocytes

LRP5 gene expression levels remained constant in monocytes treated with MV released by both LRP5‐expressing and LRP5‐silenced macrophages. Interestingly LRP5 gene levels were increased in monocytes conditioned with MV released by lipid‐loaded macrophages (Figure 5F). However, iNOS and CD80 expression levels in monocytes conditioned with MV released by untreated and lipid‐loaded macrophages in the presence or absence of LRP5 were not significantly modified (Figure 5G,H) as did the expression levels of the anti‐inflammatory genes CD163 and IL1Ra (Figure 5I,J) indicating that monocyte gene expression is unaffected by LRP5^+^MVs.

## 4 DISCUSSION

Microvesicles can stimulate targets cells by direct interaction with target receptors and the transfer of the bioactive molecules they contain.^2^, ^3^, ^7^, ^8^, ^10^, ^13^, ^15^ Here, we show, for the first time, that LRP5 is delivered into MV released by macrophages and monocytes. However, the release of LRP5^+^MV is only significantly increased in fully differentiated lipid‐loaded macrophages.

In general, macrophages are classified into two main phenotypes, classical M1 CD16^−^ activated macrophages and alternative M2 CD16^+^ activated macrophages, which regulate pro‐inflammatory and anti‐inflammatory responses, respectively.^29^ Regulation of lipid‐induced macrophage polarization is a very new field of investigation. A recent study showed that saturated fatty acid treatment induced M1‐predominant macrophages, while polyunsaturated fatty acid induced M2‐predominant macrophages.^30^ Treatment of hepatocytes with conditioned media from M1‐polarized macrophages promoted lipid synthesis and accumulation indicating that lipid‐induced macrophage M1 polarization stimulates hepatic lipid metabolism.^30^ In this study, we show that lipid‐loaded macrophages show high cell surface expression of pro‐inflammatory proteins while cell surface expression of anti‐inflammatory proteins (CD16^+^, CD163^+^ and CD206^+^) remains constantly low indicating that lipid loading induces M1 polarization. This results are supported by cell sorting experiments where isolated specific pro‐inflammatory subpopulations of macrophages release pro‐inflammatory proteins. LRP5 expression levels are higher in CD16^+^ expressing macrophages as compared to CD16^−^ macrophages. Indeed, in control conditions there is a 2.6% expression of cell surface LRP5 in CD16^−^ macrophages as compared to 16.7% in CD16^+^ macrophages. These results are in line with our previous findings where LRP5 immunofluorescent staining was increased in CD16^+^ macrophages as compared to CD16^−^ macrophages.^26^ Here, we show that LRP5 expression levels in control macrophages expressing anti‐inflammatory proteins are higher than in macrophages expressing pro‐inflammatory markers (CD16^−^, CD80^+^ and CD83^+^) indicating that in control conditions, there is more LRP5 expressed in macrophages with anti‐inflammatory phenotype. Interestingly, LRP5 cell surface expression is increased in all lipid‐loaded macrophages. Indeed, macrophages expression of pro‐inflammatory or anti‐inflammatory markers on their cell surface is independent of LRP5 expression levels, indicating that LRP5 expression is upregulated in lipid‐loaded macrophages irrespective of the macrophage inflammatory phenotype.

In the presence of extracellular lipids, there is increased release of MV. This is in line with previous studies where statin treatment (a lipid lowering agent) reduced MV shedding from platelets, endothelial cells and leukocytes carrying markers of cell activation.^31^ Similarly, decreased MV release and decreased cargo of cell activation markers after statin treatment in different cell lineages have also been described.^32^, ^33^, ^34^, ^35^, ^36^ We have explored whether MV show different inflammatory phenotypes if they are released by untreated or lipid‐loaded macrophages. Because MV are released from the cell surface of their cells of origin, we used the same pro‐inflammatory and anti‐inflammatory markers used to characterize CD16^−^ and CD16^+^ macrophages to map their released MV. Lipid‐loaded macrophages show increased release of CD16^−^MV, CD80^+^MV and CD83^+^MV, while the release of MV containing anti‐inflammatory markers (CD16^+^, CD163^+^ and CD206^+^) remained similar to untreated macrophages indicating that lipid‐loaded macrophages release MV with a pro‐inflammatory phenotype. The molecular mechanisms behind the preferential incorporation of different proteins into budding MV remain to be elucidated, but it has been suggested that it could be mediated by the cytoplasmic domains of the protein to be included into the MV.^37^

Similar to the increased cellular expression of LRP5 in lipid‐loaded CD16^−^ and CD16^+^ macrophages, lipid loading induced increased release of LRP5^+^MV in both CD16^−^MV and CD16^+^MV indicating that the expression of inflammatory markers in MV is independent of the delivery of LRP5 into MV. However, after lipid loading, only pro‐inflammatory MV were released. Therefore, only LRP5^+^CD16^−^MV, LRP5^+^CD80^+^MV and LRP5^+^CD83^+^MV were released. This raises the very interesting question of how is LRP5 delivered and released with MV containing pro‐inflammatory markers. Notably, MV production and release are stimuli and signal dependent.^38^, ^39^ For example, cytokine IL1β induces MV shedding from circulating monocytes.^40^ Accordingly, here we show that lipid stimuli induce pro‐inflammatory MV release. As lipid loaded macrophages show increased expression of LRP5 at the cell surface, it is plausible that this LRP5 will be delivered to their MV and released as LRP5^+^MV. Human macrophages expressing LRP5 have been shown to provide survival and repair to damaged tissues.^26^ It is our hypothesis that this is the function of LRP5^+^MV but further work needs to be performed to prove it.

We also explored the function of macrophage‐derived LRP5^+^MV in the polarization fate of macrophages. Lipid‐loaded macrophages that did not express LRP5 showed similar MV release than lipid‐loaded LRP5^+^ macrophages indicating that LRP5 does not participate in the MV release pathway. A reduction in LRP5^+^MV release from macrophages without LRP5 was observed.

Classically activated CD16^−^ macrophages are characterized by the expression of several pro‐inflammatory markers, including iNOS and CD80^41^, ^42^ while alternatively activated anti‐inflammatory CD16^+^ macrophages express CD163 and IL1Ra.^42^, ^43^ Treatment with MV released by macrophages devoid of LRP5 induced iNOS and CD80 expression and reduced CD163 and IL1Ra expression in naive macrophages indicating that LRP5^+^MV induce macrophages to differentiate towards an anti‐inflammatory phenotype. A limitation of this study is that the size of LRP5^+^MV was not assessed; therefore, we could not determine if LRP5^+^MV have a different size than MV devoid of LRP5. However, always the same procedure was followed to prepare MV and only MV released by control macrophages were unable to induce high expression of pro‐inflammatory genes. Also, MV released by lipid‐loaded macrophages induced increased expression of pro‐inflammatory genes independent of LRP5 expression further supporting that different stimulus in the cells of origin generates MV with different cargoes that will have different functions in the target cells.

In conclusion, here we demonstrate for the first time that a lipoprotein receptor, LRP5, is delivered into MV. MV released by lipid‐loaded macrophages contain mainly pro‐inflammatory proteins and LRP5. LRP5^+^MV induce an anti‐inflammatory genotype in naive macrophages. Therefore, a systematic blockade of monocyte/macrophage infiltration in the prevention of atherosclerosis may be less effective than originally expected if the levels of macrophage‐derived LRP5^+^MV are affected and reduced.

## CONFLICT OF INTEREST

The authors confirm that there are no conflicts of interest.

## AUTHOR CONTRIBUTION

**Aureli Luquero:** Data curation (lead); Formal analysis (equal); Investigation (equal); Methodology (lead); Software (equal); Visualization (equal); Writing‐review & editing (equal). **Gemma Vilahur:** Conceptualization (equal); Formal analysis (equal); Methodology (equal); Writing‐review & editing (equal). **Javier Crespo:** Formal analysis (supporting); Methodology (supporting); Software (equal); Writing‐review & editing (equal). **Lina Badimon:** Conceptualization (lead); Funding acquisition (lead); Methodology (equal); Resources (equal); Supervision (equal); Writing‐review & editing (equal). **Maria Borrell‐Pages:** Conceptualization (supporting); Data curation (equal); Formal analysis (equal); Funding acquisition (supporting); Investigation (lead); Methodology (equal); Supervision (lead); Writing‐original draft (lead); Writing‐review & editing (lead).

## Supporting information

Fig S1

Fig S2

Fig S3

Table S1

## Data Availability

The data underlying this article are available in the article and in its online supplementary material.

## References

[1] Ridger VC, Boulanger CM, Angelillo‐Scherrer A, et al. Microvesicles in vascular homeostasis and diseases position paper of the European society of cardiology (ESC) working group on atherosclerosis and vascular biology. Thromb Haemost. 2017;117:1296‐1316. 28569921 10.1160/TH16-12-0943

[2] Mathivanan S, Ji H, Simpson RJ. Exosomes: extracellular organelles important in intercellular communication. J Proteomics. 2010;73:1907‐1920. 20601276 10.1016/j.jprot.2010.06.006

[3] Camussi G, Deregibus MC, Bruno S, Cantaluppi V, Biancone L. Exosomes/microvesicles as a mechanism of cell‐to‐cell communication. Kidney Int. 2010;78:838‐848. 20703216 10.1038/ki.2010.278

[4] Taylor DD, Gercel‐Taylor C. Exosomes/microvesicles: mediators of cancer‐associated immunosuppressive microenvironments. Semin Immunopathol. 2011;33:441‐454. 21688197 10.1007/s00281-010-0234-8

[5] Cocucci E, Racchetti G, Meldolesi J. Shedding microvesicles: artefacts no more. Trends Cell Biol. 2009;19:43‐51. 19144520 10.1016/j.tcb.2008.11.003

[6] Février B, Raposo G. Exosomes: endosomal‐derived vesicles shipping extracellular messages. Curr Opin Cell Biol. 2004;16:415‐421. 15261674 10.1016/j.ceb.2004.06.003

[7] Hunter MP, Ismail N, Zhang X, et al. Detection of microRNA expression in human peripheral blood microvesicles. PLoS One. 2008;3:e3694. 19002258 10.1371/journal.pone.0003694 PMC2577891

[8] Ratajczak J, Wysoczynski M, Hayek F, Janowska‐Wieczorek A, Ratajczak MZ. Membrane‐derived microvesicles: important and underappreciated mediators of cell‐to‐cell communication. Leukemia. 2006;20:1487‐1495. 16791265 10.1038/sj.leu.2404296

[9] Valadi H, Ekström K, Bossios A, Sjöstrand M, Lee JJ, Lötvall JO. Exosome‐mediated transfer of mRNAs and microRNAs is a novel mechanism of genetic exchange between cells. Nat Cell Biol. 2007;9:654‐659. 17486113 10.1038/ncb1596

[10] Baj‐Krzyworzeka M, Szatanek R, Węglarczyk K, et al. Tumour‐derived microvesicles carry several surface determinants and mRNA of tumour cells and transfer some of these determinants to monocytes. Cancer Immunol Immunother. 2006;55:808‐818. 16283305 10.1007/s00262-005-0075-9 PMC11030663

[11] MacKenzie A, Wilson HL, Kiss‐Toth E, Dower SK, North RA, Surprenant A. Rapid secretion of interleukin‐1β by microvesicle shedding. Immunity. 2001;15:825‐835. 11728343 10.1016/s1074-7613(01)00229-1

[12] Frey B, Munoz LE, Pausch F, et al. The immune reaction against allogeneic necrotic cells is reduced in Annexin A5 knock out mice whose macrophages display an anti‐inflammatory phenotype. J Cell Mol Med. 2009;13:1391‐1399. 18624762 10.1111/j.1582-4934.2008.00395.x PMC4496152

[13] Martínez MC, Larbret F, Zobairi F, et al. Transfer of differentiation signal by membrane microvesicles harboring hedgehog morphogens. Blood. 2006;108:3012‐3020. 16778137 10.1182/blood-2006-04-019109

[14] Ratajczak J, Miekus K, Kucia M, et al. Embryonic stem cell‐derived microvesicles reprogram hematopoietic progenitors: evidence for horizontal transfer of mRNA and protein delivery. Leukemia. 2006;20:847‐856. 16453000 10.1038/sj.leu.2404132

[15] Collino F, Deregibus MC, Bruno S, et al. Microvesicles derived from adult human bone marrow and tissue specific mesenchymal stem cells shuttle selected pattern of miRNAs. PLoS One. 2010;5:e11803. 20668554 10.1371/journal.pone.0011803 PMC2910725

[16] Montecalvo A, Larregina AT, Shufesky WJ, et al. Mechanism of transfer of functional microRNAs between mouse dendritic cells via exosomes. Blood. 2012;119:756‐766. 22031862 10.1182/blood-2011-02-338004 PMC3265200

[17] Arderiu G, Peña E, Badimon L. Angiogenic microvascular endothelial cells release microparticles rich in tissue factor that promotes postischemic collateral vessel formation. Arterioscler Thromb Vasc Biol. 2015;35:348‐357. 25425620 10.1161/ATVBAHA.114.303927

[18] Boulanger CM, Loyer X, Rautou PE, Amabile N. Extracellular vesicles in coronary artery disease. Nat Rev Cardiol. 2017;14:259‐272. 28150804 10.1038/nrcardio.2017.7

[19] Badimon L, Vilahur G. Thrombosis formation on atherosclerotic lesions and plaque rupture. J Intern Med. 2014;276:618‐632. 25156650 10.1111/joim.12296

[20] Borrell‐Pages M, Romero JC, Juan‐Babot O, Badimon L. Wnt pathway activation, cell migration, and lipid uptake is regulated by low‐density lipoprotein receptor‐related protein 5 in human macrophages. Eur Heart J. 2011;32:2841‐2850. 21398644 10.1093/eurheartj/ehr062

[21] Clevers H, Nusse R. Wnt/β‐catenin signaling and disease. Cell. 2012;149:1192‐1205. 22682243 10.1016/j.cell.2012.05.012

[22] Borrell‐Pages M, Vilahur G, Romero JC, Casaní L, Bejar MT, Badimon L. LRP5/canonical Wnt signalling and healing of ischemic myocardium. Basic Res Cardiol. 2016;111:67. 27704249 10.1007/s00395-016-0585-y

[23] Badimon L, Casaní L, Camino‐Lopez S, Juan‐Babot O, Borrell‐Pages M. GSK3β inhibition and canonical Wnt signaling in mice hearts after myocardial ischemic damage. PLoS One. 2019;14:e0218098. 31220102 10.1371/journal.pone.0218098 PMC6586285

[24] Mosser DM, Edwards JP. Exploring the full spectrum of macrophage activation. Nat Rev Immunol. 2008;8:958‐969. 19029990 10.1038/nri2448 PMC2724991

[25] Murray PJ, Wynn TA. Protective and pathogenic functions of macrophage subsets. Nat Rev Immunol. 2011;11:723‐737. 21997792 10.1038/nri3073 PMC3422549

[26] Borrell‐Pages M, Romero JC, Crespo J, Juan‐Babot O, Badimon L. LRP5 associates with specific subsets of macrophages: molecular and functional effects. J Mol Cell Cardiol. 2016;90:146‐156. 26666179 10.1016/j.yjmcc.2015.12.002

[27] Borrell‐Pagès M, Romero JC, Badimon L. LRP5 negatively regulates differentiation of monocytes through abrogation of Wnt signalling. J Cell Mol Med. 2014;18:314‐325. 24266894 10.1111/jcmm.12190 PMC3930418

[28] Badimon L, Luquero A, Crespo J, Peña E, Borrell‐Pages M. PCSK9 and LRP5 in macrophage lipid internalization and inflammation. Cardiovasc Res. 2020;cvaa254. 10.1093/cvr/cvaa254 32991689

[29] Tacke F, Zimmermann HW. Macrophage heterogeneity in liver injury and fibrosis. J Hepatol. 2014;60:1090‐1096. 24412603 10.1016/j.jhep.2013.12.025

[30] Wu HM, Ni XX, Xu QY, Wang Q, Li XY, Hua J. Regulation of lipid‐induced macrophage polarization through modulating peroxisome proliferator‐activated receptor‐gamma activity affects hepatic lipid metabolism via a Toll‐like receptor 4/NF‐κB signaling pathway. J Gastroenterol Hepatol. 2020;35:1998‐2008. 32128893 10.1111/jgh.15025

[31] Suades R, Padró T, Alonso R, Mata P, Badimon L. Lipid‐lowering therapy with statins reduces microparticle shedding from endothelium, platelets and inflammatory cells. Thromb Haemost. 2013;110:366‐377. 23740299 10.1160/TH13-03-0238

[32] Nomura S, Shouzu A, Omoto S, et al. Effects of eicosapentaenoic acid on endothelial cell‐derived microparticles, angiopoietins and adiponectin in patients with type 2 diabetes. J Atheroscler Thromb. 2009;16:83‐90. 19403992 10.5551/jat.e091

[33] Nomura S, Inami N, Shouzu A, Rase F, Maeda Y. The effects of pitavastatin, eicosapentaenoic acid and combined therapy on platelet‐derived microparticles and adiponectin in hyperlipidemic, diabetic patients. Platelets. 2009;20:406‐414. 19172517 10.1080/09537100802409921

[34] Tramontano AF, O’Leary J, Black AD, Muniyappa R, Cutaia MV, El‐Sherif N. Statin decreases endothelial microparticle release from human coronary artery endothelial cells: implication for the Rho‐kinase pathway. Biochem Biophys Res Commun. 2004;320:34‐38. 15207698 10.1016/j.bbrc.2004.05.127

[35] Sommeijer DW, Joop K, Leyte A, Reitsma PH, Ten Cate H. Pravastatin reduces fibrinogen receptor gpIIIa on platelet‐derived microparticles in patients with type 2 diabetes. J Thromb Haemost. 2005;3:1168‐1171. 15946206 10.1111/j.1538-7836.2005.01403.x

[36] Mobarrez F, He S, Bröijersen A, et al. Atorvastatin reduces thrombin generation and expression of tissue factor, p‐selectin and GPIIIa on platelet‐derived microparticles in patients with peripheral arterial occlusive disease. Thromb Haemost. 2011;106:344‐352. 21614411 10.1160/TH10-12-0810

[37] Barclay AN, Brown M, Law SKA, McKnight A, Tomlinson M, Van der Merwe P. The Leucocyte Antigen Factsbook. Oxford, UK: Elsevier, Academic Press; 1997:231.

[38] Williams C, Palviainen M, Reichardt NC, Siljander PRM, Falcón‐Pérez JM. Metabolomics applied to the study of extracellular vesicles. Metabolites. 2019;9:276. 10.3390/metabo9110276 PMC6918219 31718094

[39] Turchinovich A, Drapkina O, Tonevitsky A. Transcriptome of extracellular vesicles: state‐of‐the‐art. Front Immunol. 2019;10:202. 30873152 10.3389/fimmu.2019.00202 PMC6404625

[40] Ward JR, West PW, Ariaans MP, et al. Temporal interleukin‐1β secretion from primary human peripheral blood monocytes by P2X7‐independent and P2X7‐dependent mechanisms. J Biol Chem. 2010;285:23147‐23158. 20495003 10.1074/jbc.M109.072793 PMC2906308

[41] Xue Q, Yan Y, Zhang R, Xiong H. Regulation of iNOS on immune cells and its role in diseases. Int J Mol Sci. 2018;19:3805. 10.3390/ijms19123805 PMC6320759 30501075

[42] Bertani FR, Mozetic P, Fioramonti M, et al. Classification of M1/M2‐polarized human macrophages by label‐free hyperspectral reflectance confocal microscopy and multivariate analysis. Sci Rep. 2017;7:8965. 28827726 10.1038/s41598-017-08121-8 PMC5566322

[43] Alvarado‐Vazquez PA, Bernal L, Paige CA, et al. Macrophage‐specific nanotechnology‐driven CD163 overexpression in human macrophages results in an M2 phenotype under inflammatory conditions. Immunobiology. 2017;222:900‐912. 28545809 10.1016/j.imbio.2017.05.011 PMC5718187

