# Supplementary figures and images for: Microvesicles carrying LRP5 induce macrophage polarization to an anti‐inflammatory phenotype
Source: J Cell Mol Med. 2021 Jul 19;25(16):7935–47. doi: 10.1111/jcmm.16723 (PMC8358886; doi:10.1111/jcmm.16723)

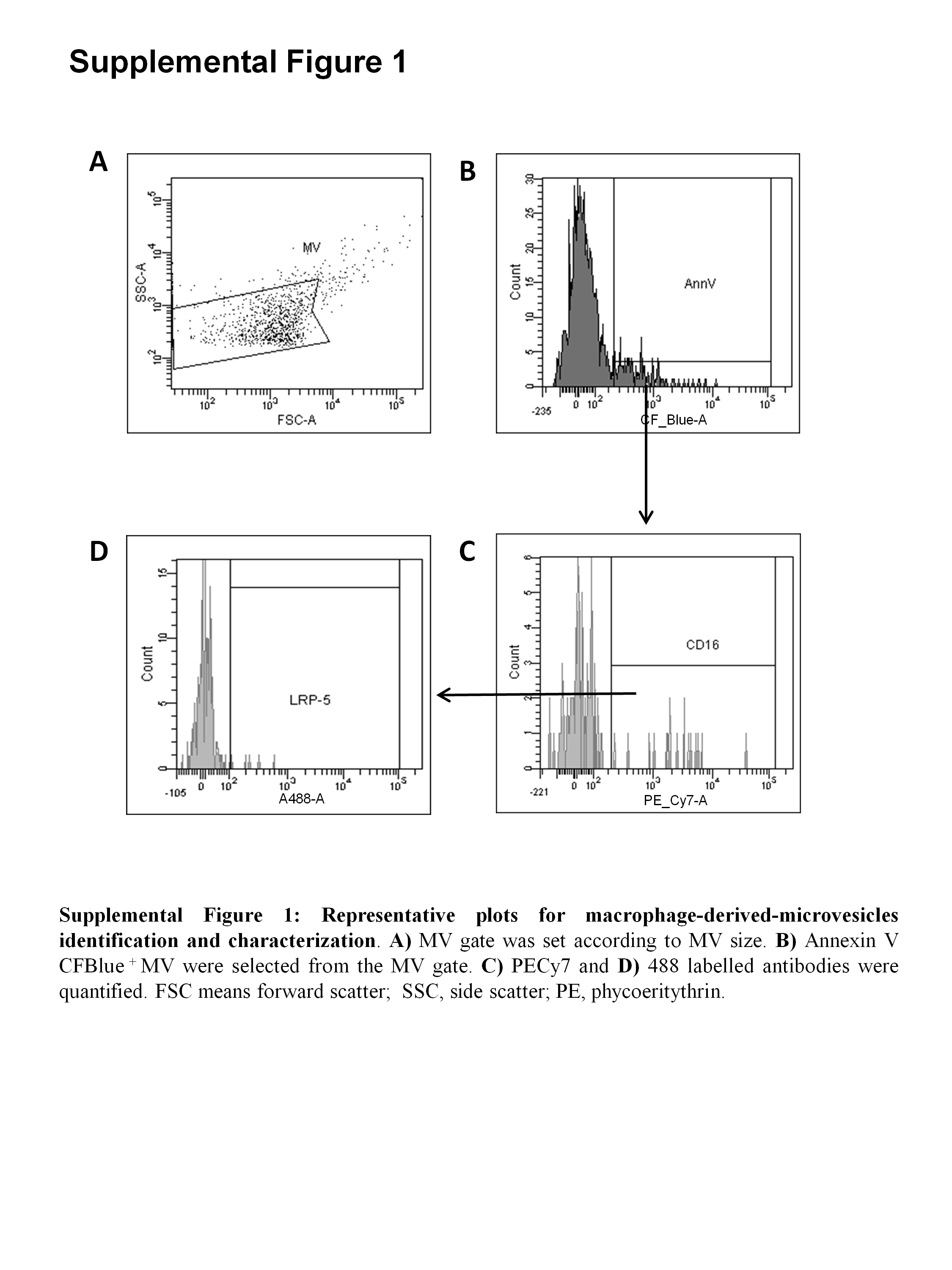

Supplement: Supplementary file 1 — Fig S1 [file JCMM-25-7935-s004.TIF]

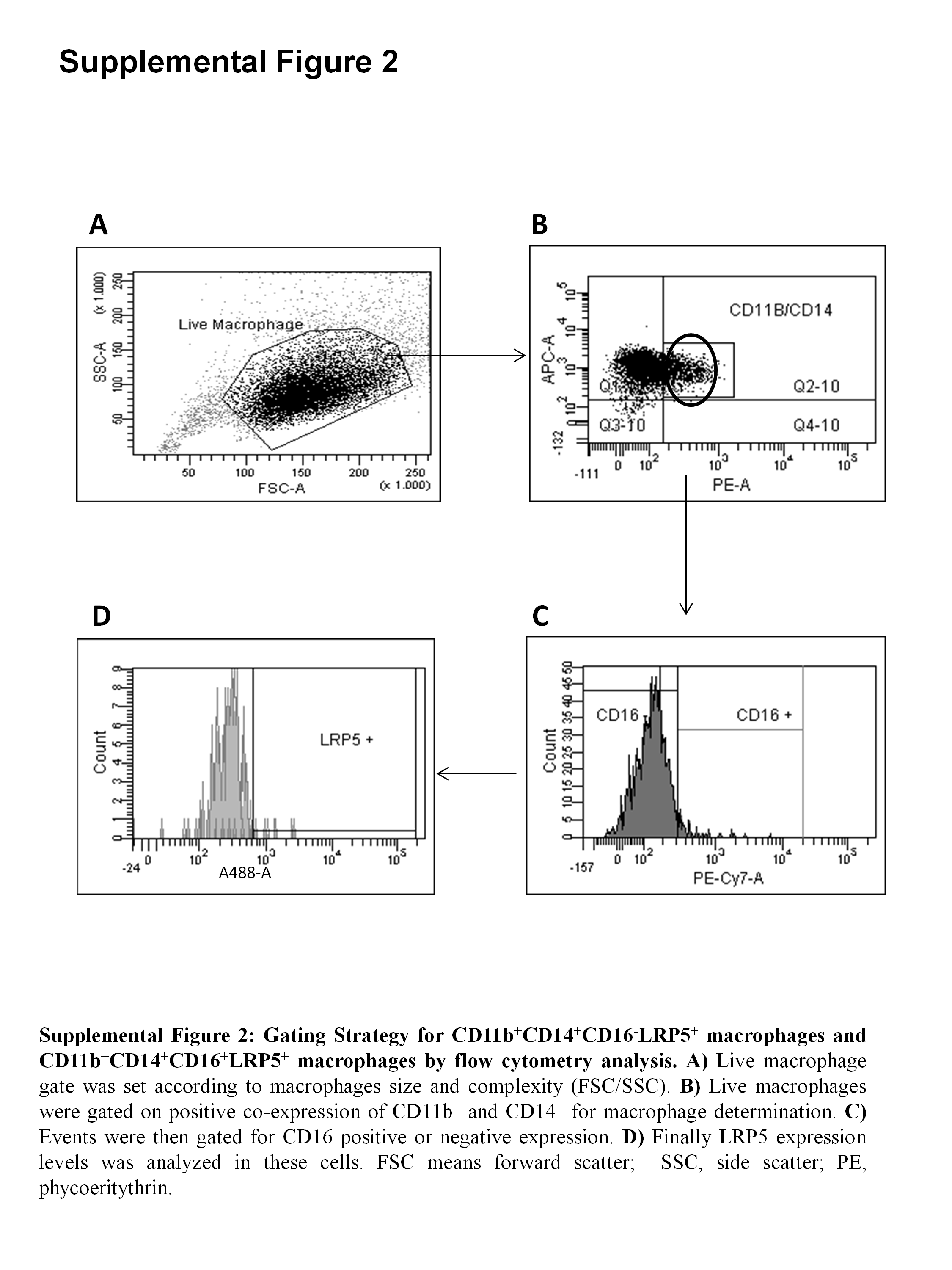

Supplement: Supplementary file 2 — Fig S2 [file JCMM-25-7935-s001.TIF]

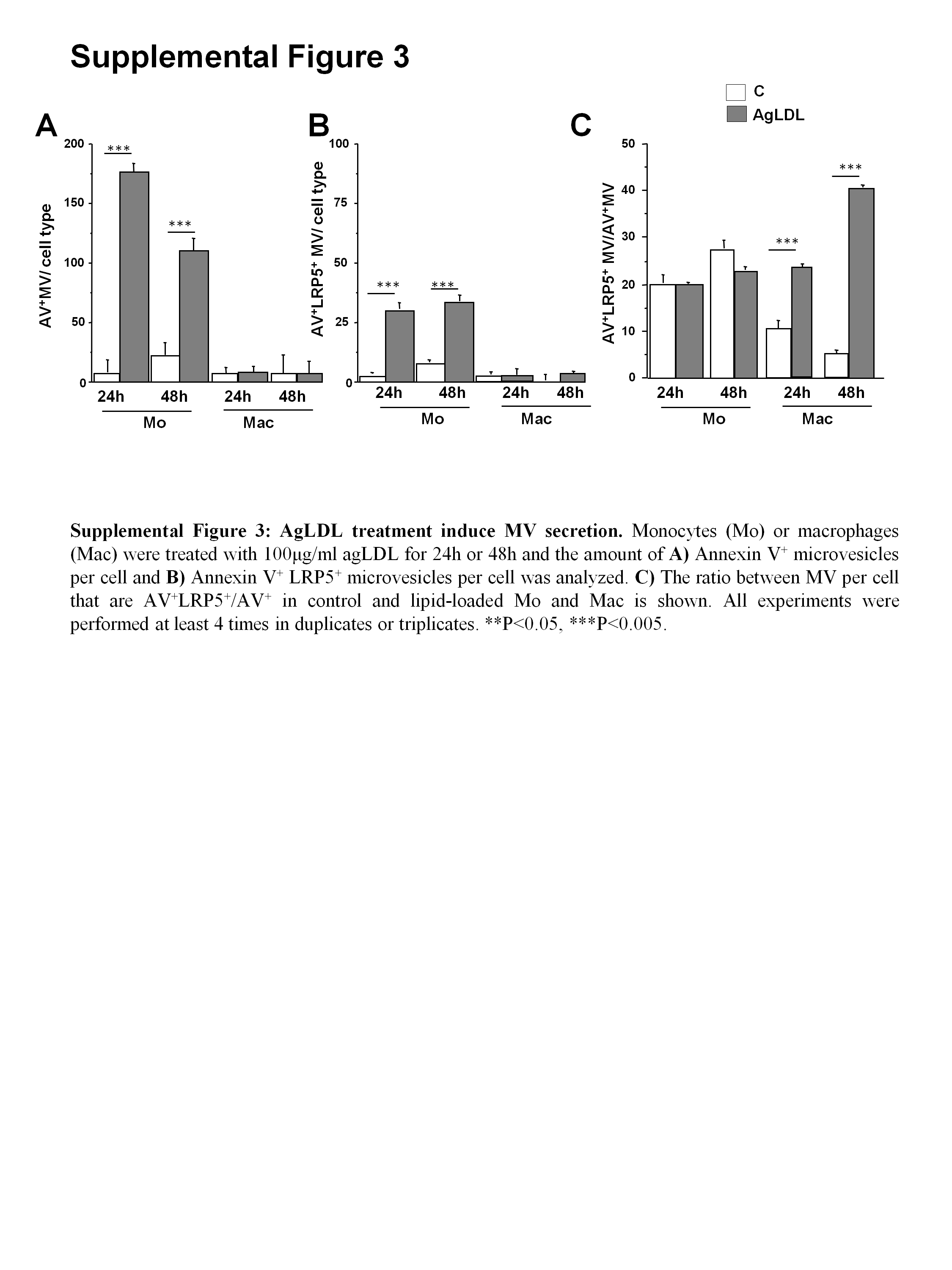

Supplement: Supplementary file 3 — Fig S3 [file JCMM-25-7935-s002.TIF]
