# Supplementary material for: Microvesicles carrying LRP5 induce macrophage polarization to an anti‐inflammatory phenotype
Source: J Cell Mol Med. 2021 Jul 19;25(16):7935–47. doi: 10.1111/jcmm.16723 (PMC8358886; doi:10.1111/jcmm.16723)
Supplement: Supplementary file 4 — Table S1 [file JCMM-25-7935-s003.docx]

**Supplemental Tables**

| Supplemental Table I. Cell molecules for circulating microvesicle identification and characterization**.** | | | | | |  |
| --- | --- | --- | --- | --- | --- | --- |
|  |  |  |  |  |  |  |
| **mAb** | **Alternative name** | **Conjugation** | **Clone** | **µL/test** | **Company** | **Reference** |
| **CD16** | Fc gamma RIII | PE-Cyanine7 | eBioCB16 | 2,5 | Invitrogen | 25-0168-42 |
| **CD206** | Macrophage mannose receptor | PE-Cyanine7 | 19.2 | 2,5 | Invitrogen | 25-2069-42 |
| **CD163** | Haemoglobin-Heptaglobin complex Receptor | PE-Cyanine7 | eBioGHI/61 (GHI/61) | 5 | Invitrogen | 25-1639-42 |
| **CD80** | B7-1 Membrane Protein | Super Bright 436 | 2D10.4 | 5 | Invitrogen | 62-0809-42 |
| **CD83** | B-cell activation protein | PerCP-eFluo 710 | HB15e | 5 | Invitrogen | 46-0839-42 |
| **LRP5** | LRP5 | 488 | Polyclonal | 1 | Bioss | bs-411R |
| **AV** | PS- Binding protein | CF Blue | --- | 5 | Immunostep | ANVCFB-200 |
|  |  |  |  |  |  |  |

mAb indicates monoclonal antibody; PE, phycoerithrin; AV Annexin V; PS, phosphatidylserine.

| Supplemental Table II. Cell molecules for macrophage identification and characterization**.** | | | | |  |  |
| --- | --- | --- | --- | --- | --- | --- |
|  |  |  |  |  |  |  |
| **mAb** | **Alternative name** | **Conjugation** | **Clone** | **µL/test** | **Company** | **Reference** |
| **CD16** | Fc gamma RIII | PE-Cyanine7 | eBioCB16 | 5 | Invitrogen | 25-0168-42 |
| **CD206** | Macrophage mannose receptor | PE-Cyanine7 | 19.2 | 5 | Invitrogen | 25-2069-42 |
| **CD163** | Haemoglobin-Heptaglobin complex Receptor | PE-Cyanine7 | eBioGHI/61 (GHI/61) | 5 | Invitrogen | 25-1639-42 |
| **CD11b** | Macrophage-1 Antigen (Mac-1) | PE | M1/70 | 5 | Invitrogen | 12-0112-82 |
| **CD14** | LPS Receptor | PerCPCyanine5.5 | Sa2-8 | 5 | Invitrogen | 45-0141-82 |
| **CD14** | LPS Receptor | PE | 61D3 | 5 | Invitrogen | 12-0149-42 |
| **CD80** | B7-1 Membrane Protein | Super Bright 436 | 2D10.4 | 5 | Invitrogen | 62-0809-42 |
| **CD83** | B-cell activation protein | PerCP-eFluor710 | HB15e | 5 | Invitrogen | 46-0839-42 |
| **LRP5** | LRP 5 | 488 | Polyclonal | 1 | Bioss | bs-411R |

mAb indicates monoclonal antibody; PE, phycoerithrin; APC, allophycocyanin; LPS, lipopolysaccharide.
